# Cytokine-mediated crosstalk between cancer stem cells and their inflammatory niche from the colorectal precancerous adenoma stage to the cancerous stage: Mechanisms and clinical implications

**DOI:** 10.3389/fimmu.2022.1057181

**Published:** 2022-11-17

**Authors:** Guanglin Cui, Ziqi Wang, Hanzhe Liu, Zhigang Pang

**Affiliations:** ^1^ Research Group of Gastrointestinal Diseases, The Second Affiliated Hospital of Zhengzhou University, Zhengzhou, Henan, China; ^2^ Faculty of Health Science, Nord University, Levanger, Norway; ^3^ College of Medical Imaging, Mudanjiang Medical University, Mudanjiang, China; ^4^ School of Stomatology, Wuhan University, Wuhan, China

**Keywords:** cytokine, colorectum, tumorigenesis, stem cell, cancer stem cells

## Abstract

The majority of colorectal cancers (CRCs) are thought to arise from precancerous adenomas. Upon exposure to diverse microenvironmental factors, precancerous stem cells (pCSCs) undergo complex genetic/molecular changes and gradually progress to form cancer stem cells (CSCs). Accumulative evidence suggests that the pCSC/CSC niche is an inflammatory dominated milieu that contains different cytokines that function as the key communicators between pCSCs/CSCs and their niche and have a decisive role in promoting CRC development, progression, and metastasis. In view of the importance and increasing data about cytokines in modulating pCSCs/CSC stemness properties and their significance in CRC, this review summarizes current new insights of cytokines, such as interleukin (IL)-4, IL-6, IL-8, IL-17A, IL-22, IL-23, IL-33 and interferon (IFN)-γ, involving in the modulation of pCSC/CSC properties and features in precancerous and cancerous lesions and discusses the possible mechanisms of adenoma progression to CRCs and their therapeutic potential.

## Introduction

Globally, colorectal cancer (CRC) is the fourth most frequent cancer and it seriously endangers the public health because of its high incidence and mortality. According to data from 2020, 1.8 million newly diagnosed cases and 0.8 million deaths were estimated worldwide (1). Although the precise mechanism for CRC development is not fully clear, numerous clinical and basic studies have strongly suggested that the development of CRC is mostly a result of a stepwise process of histological, genetic, molecular, and immunological alterations in precancerous adenomas over time (2). Clinical observations also revealed that only a small number of adenomas eventually progress to CRCs and most adenomas maintain a relatively stable status for years (3). Furthermore, although the exact mechanisms by which various factors determine the progression of precancerous adenoma to CRC remain incompletely resolved, extensive evidence suggests that cancers originally develop from a subpopulation of tumor-initiating cells, termed cancer stem cells (CSCs) (4). CSCs are involved not only in tumor initiation, but also in tumor progression, invasion, metastasis, and resistance to anticancer therapies (5). Precancerous stem cells, referred to as pCSCs, in adenomatous lesions may have the potential for bidirectional differentiation into both benign and malignant lesions, depending on their niche (6). Studies have shown that the interaction between pCSCs and their niche has a decisive role in directing the differentiation of pCSCs to CSCs, which initiates the transitional process of adenoma to CRC (7). Currently, significant efforts have been made toward the evaluation of modulatory factors’ effects on pCSCs/CSCs within the tumor microenvironment (TME) (8).

The adenoma/CRC TME is an inflammatory milieu, in which several types of immune cells are recruited, activated, and then undergo phenotypic and functional changes (9). Accumulated data show that these immune cells together with other types of cells, e.g., tumor cells, stromal cells, mesenchymal stem cells (MSCs) and tumor endothelial cells, produce large amounts of cytokines that play multiple modulatory roles in immunosuppression regulation, tumor cell growth and angiogenesis processes during colorectal carcinogenesis. The overexpression of cytokines has been shown to be closely associated with adenoma progression and CRC metastasis (2). Emerging evidence indicates that stem cells (SCs) in both physiological and pathological conditions deeply interact with their inflammatory niche (10), in which cytokines may function as the main communicators to control the CSC properties and features that are strongly linked to the recurrence and metastasis of CRC (11). Therefore, we hypothesize that one of the potential mechanisms by which cytokines can promote the development of CRC is *via* the modulation of pCSCs/CSCs from colorectal precancerous adenomas to CRCs.

In view of the importance and increasing data about cytokines as communicators in mediating crosstalk between pCSCs/CSCs and their surrounding niche, understanding this modulatory effect is essential to exploit the functional mechanisms of pCSCs/CSCs as their translation from precancerous lesions to cancerous lesions. Thus, this review will summarize recent advances in the research field of cytokines involved in the modulation of pCSC/CSC properties and functions. In addition, the possible mechanisms of the progression of pCSCs to CSCs and their therapeutic potential are discussed.

## pCSCs/CSCs in the adenoma and CRC tissues

Previous studies have revealed that pCSCs/CSCs can be identified in both precancerous and cancer lesions (12). By using reverse transcription-polymerase chain reaction and RNA *in-situ* hybridization techniques, Jang et al. (13) evaluated the expression of various SC markers, e.g., leucine-rich repeat-containing G-protein-coupled receptor 5 (LGR5), ASCL2, EPHB2 and olfactomedin-4 (OLFM4) in the precancerous lesions of CRC. In the normal colonic mucosa, hyperplastic polyps, and sessile serrated adenomas, SCs positive for these markers were distributed in the base region of colonic crypts. However, the distribution of SCs in adenomas was presented as a diffuse pattern in the adenomatous epithelium (13). Another study by Baker et al. (14) also showed that conventional colorectal adenomas diffusely expressed high levels of LGR5. In contrast, serrated lesions displayed basal localization of LGR5 as seen in a normal crypt. Bartley and colleagues (15) used aldehyde dehydrogenase isoform 1A1 (ALDH1A1) as a marker to identify SCs in precancerous colorectal adenomas and found that adenomas located in the right site of the colon tended to express a higher level of ALDH1A1 labeling indices than those in the left site of the colon.

Furthermore, a study by Humphries et al. (16) used a combination of nuclear and mitochondrial DNA lesions and epigenetic markers to track the lineage of SCs in human adenomas and identified SCs in precancerous adenomas. They showed that new growth of intratumor clones of SCs occurred infrequently, suggesting that SCs in precancerous adenomas were mostly in a stable condition (16).

We have previously used a various set of cell markers, such as Musashi, CD133, LGR5 and ALDH1, as biomarkers to examine the presentation of SCs in different compartments of adenomas (17). We found that populations of SCs labeled by diverse markers were increased in the adenoma tissues. In addition, a spatial change in SCs in the adenoma was observed: SCs expanded from the base to the middle part of the transitional crypt and final finally reached the surface of the adenoma, reflecting a changed track pathway of SCs during the development of adenomas. Further analysis revealed that increased populations of LGR5 and aldehyde dehydrogenase 1 (ALDH1) labeled SCs in the adenoma were associated with the degree of dysplasia, suggesting the involvement of SCs in the establishment of adenomas (17).

In a very elegant study, Gao’s group has experimentally identified a new type of SC that has the potential for both benign and malignant differentiation in colorectal precancerous lesions and named these SCs ‘pCSCs’ (6). These pCSCs have the features of both normal SCs and CSCs and are potentially regulated by the stem-cell protein Piwil2 (6), which is widely expressed by cancer cells and precancerous cells in various types of cancers (18–23). Carpentino et al. found that ALDH1-positive pCSCs were involved in the transition of colitis to CRC in xenografting studies as well as *in vitro* (24), which was accompanied by upregulated expression of interleukin (IL)-6 and IL-8 in the TME (24). Therefore, exploring the mechanism by which pCSCs progress to CSCs is critically important for understanding CRC development mechanisms, and targeting pCSCs may have an important translational significance in blocking the transition of precancerous lesions to cancerous lesions.

Similarly, the evaluation of CSCs in CRC has also been conducted. CSCs labeled by various markers, e.g., Musashi, CD44, CD133, CD166, epithelial cell adhesion molecules, LGR5 and ALDH1, were observed in the CRC epithelium (13–15, 17, 25–30). Further studies showed that the density of CSCs was associated with the progression, metastasis, and prognosis of patients with CRC (30–34). We tracked the temporal and spatial changes of CSCs in CRC tissues and found that CSCs were moving up from the basal to the middle region inside the adjacent transitional crypts and finally reached the surface of CRC epithelium, with an increased population (17). Recently, studies reported that CSCs increased the tumor’s ability to resist chemotherapy or immunotherapy (33, 35, 36), indicating that CSCs are as an effective target for improving the treatment of CRCs (37, 38).

The regulation of CSC properties and features is an important research field. Compelling evidence has shown that that both pCSCs in adenomas and CSCs in CRCs live in a milieu that contains dense immune cells and stromal cells (11, 39–46); these cells produce high amounts of cytokines and form an inflammatory niche that modulates pCSCs renewal, differentiation and the early onset of cancer in a coordinated manner (8, 44–46). Emerging research has shown that cytokines are involved in the modulation of CSCs (11). Therefore, it is necessary to evaluate cytokines in the surrounding niche that can specifically affect the properties and features of CSCs and determine the recurrence and metastasis of CRC.

## Changes in the cytokine profile in the TME along the colorectal adenoma-carcinoma sequence

It is well known that the adenoma TME contains high densities of immune cells that produce large amounts of cytokines (2). These cytokines have complex functions by modulating immune function and interacting with premalignant adenoma cells, which determines whether premalignant lesions remain stable or progress (2). For example, we have demonstrated that the levels of interferon (IFN)-γ, IL-12A and IL-18 were increased in adenoma tissues and decreased in CRC tissues (47). Since these three cytokines have been recognized as critical factors in maintaining antitumor immunity, the increasing IFN-γ, IL-12 and IL-18 in the TME may imply an initial effort of the host to combat the appearance of adenomas in the colorectal mucosa. Importantly, IFN-γ is a cytokine that plays a critical role in controlling the survival of CSCs (48). Increased IFN-γ levels at the adenoma stage may help the host immunity to selectively eliminate pCSCs sensitive to immunosurveillance and allow pCSCs resistant to immunosurveillance to survive and progress to CSCs.

From the adenoma stage to the CRC stage, tumor-promoting relevant cytokines, such as IL-6, IL-8, IL-17A and IL-33, are significantly increased (49–52). These cytokines have been shown to stimulate CRC invasion and metastasis by inducing immunosuppression and enhancing angiogenesis (53, 54). Mechanistically, cytokine-promoted CRC progression occurs through the activation of diverse signaling pathways (41). For example, IL-4 stimulation induced the activation of signal transducer and activator of transcription (STAT)-6 phosphorylation in epithelial cells (55). Enhanced STAT-6 phosphorylation in patients with CRC was correlated with an advanced stage and decreased survival (56). Animal experiments showed that IL-6 promoted the proliferation of CRC cells and the progression of both inflammation-related and sporadic CRCs *via* the activation of STAT-3 signaling in mice (57, 58). Similarly, IL-17A could indirectly activate STAT-3 through IL-6.

Furthermore, the promoting effect of IL-8 on cancer cell proliferation, invasion, and angiogenesis occurred through activation of the Akt and MAPK signaling pathways (59). The nuclear factor kappa B (NF-κB) signaling pathway is also a key regulator of cancer cell proliferation, progression and metastasis, and activation of NF-κB protein cascade complexes by cytokines has been observed in patients with CRC (60). Somone et al. (61) demonstrated that the stimulatory effect of TH17 cytokines, TNF-α and IL-6, could synergistically activate the NF-κB signaling pathway and then promote CRDC cell growth. Their results also showed that IL-22 and IL-6 could activate STAT-3 signaling.

Interestingly, several studies have revealed that the activation of these signaling pathways contributes to the stemness of CSCs in CRC. For example, persistent action of STAT-3 was correlated with enhanced stemness and proliferation of CRC cells (58). Additionally, aberrant NF-κB signaling has been identified in colon cancer cells, which was regulated by downregulating miR-195-5p/497–5p and upregulating MCM2 (62). These data suggest that the modulatory effect of cytokines on CSCs may occur *via* the activation of diverse signaling pathways in CSCs, targeting signaling pathways by specific inhibitors may block the effect of cytokines (63).

It must be noted that cytokines with anticancer capacity have been reported. For example, studies have shown that IL-12 produced from mature dendritic cells is a strong inducer of the antitumor-specific immune response (64), and IFN-γ, secreted by T helper (TH)-1 cells, cytotoxic T cells and nature killer cells, plays a key role in the activation of anticancer cellular immunity and the induction of tumor cell apoptosis (65). Studies have revealed that decreased levels of IL-12 and IFN-γ in patients with CRC were associated with CRC progression (47, 65), indicating they have a critical modulatory effect on anticancer immunity.

The potential of cytokine-based immunotherapy in patients with CRC has been intensively evaluated. For example, a study by Ying et al. (66) showed that blocking IL-6 signaling with anti-IL-6 or anti-IL-6 receptor antibodies suppressed STAT-3 signaling pathway and enhanced the efficacy of anticancer drugs *in vitro*. Furthermore, a phase I/II clinical trial with siltuximab (anti-IL-6 monoclonal antibody) monotherapy that included 32 CRC patients showed that patients tolerated it well at different doses. Studies have also shown the benefits of a combination of anti-PD-1 with anti-IL-17 therapy in microsatellite stable (MSS) CRC (67), and blocking IL-17 signaling may significantly enhance the response to anti-PD1 treatment both *in vitro* and *in vivo* (68). In addition, studies have demonstrated that cytokines were associated with resistance to therapies in CRC (69–72). Therefore, targeting these cytokine or receptor signals, e.g., IL-17A, may have significant clinical potential and improve the response to therapeutics and this has been considered a promising immunotherapeutic strategy (68, 73, 74).

## Role of cytokines as communicators in mediating crosstalk between CSCs and their niche

Persistent exposure of the niche to proinflammatory cytokines may significantly influence the biological features and functions of CSCs in CRC. In the following paragraphs, we briefly summarize and discuss the significance of cytokines as the main communicators in mediating the interaction between CSCs and their niche.

### IL-4

IL-4 is a multifunctional cytokine that plays a critical role in the regulation of host antitumor immunity in diverse types of cancers. Recently, the modulatory effect of IL-4 on CSCs has been identified (75, 76). Research data indicated that IL-4 could potentially stimulate stemness genes and CSC survival, which were associated with cancer metastasis, recurrence, and drug-resistance (77–81). Abrogation of IL-4 signaling with an IL-4Rα antagonist or anti-IL-4 neutralizing antibody could strongly inhibit the survival of colon CSCs and block resistance to chemotherapeutic drugs (82). Interestingly, studies also revealed that IL-4 could function as an autocrine factor to participate in the regulation of CSCs and the induction of drug resistance (82).

### IL-6

IL-6 is an inflammatory cytokine produced by several types of cells e.g., T lymphocytes, macrophages, adenoma/CRC cells, and surrounding stromal cells (83). The current scientific evidence suggests that IL-6 and its functional receptor IL-6R are involved in the pathogenesis of CRC development, progression, and metastasis (84, 85). Previous studies have shown that IL-6 can activate STAT-3, the major downstream signaling pathway for IL-6, and it plays a role in the maintenance of CRC cell survival and tumor initiation (86, 87). JAK-STAT signaling is highly active in CSCs, and studies have reported that activation of STAT-3 by IL-6 has a profound effect on tumor initiation and progression, invasion and metastasis by protecting tumor cells from apoptosis, driving epithelial–mesenchymal plasticity, and enhancing angiogenesis (86, 88, 89). In addition, IL-6//STAT-3 is involved in the induction of drug resistance in CRC (86, 88, 89).

Notch is another important oncogenic signaling pathway that contributes to metastasis by inducing epithelial–mesenchymal transition (EMT) in CRC (90). Studies have shown that the activation of Notch1 leads to changes in CSC phenotypes and properties (91, 92). The interplay between IL-6 and Notch has been studied, in which IL-6 was one of the main inducers of the activation of Notch signaling in both MSCs (93) and CSCs (94), and it controbuted to the induction of metastasis and drug resistance (94). Thus, a regulatory effect of IL-6 on CSCs has been postulated. Indeed, Wang et al. (95) reported that IL-6 induced the deacetylation of FRA1 at the Lys-116 residue located within its DNA-binding domain and promoted CRC stem-like properties by affecting transcriptional and posttranslational regulation.

A study by Ying et al. (66) demonstrated that the expression of Notch 3 was significantly upregulated in colon cancer spheroid-forming cells compared with adherent cells, which was upregulated by IL-6 administration, and blocked by an anti-human IL-6R monoclonal antibody. Furthermore, they confirmed that blocking the IL-6 receptor or Notch3 inhibition may be superior to STAT-3 inhibition for CSC-targeting therapies concomitant with anticancer drugs in CRC cell lines (66). Kim et al. (96) found that IL-6 and IL-8 produced by stromal myofibroblasts could expand CSCs by activating Notch/HES1 and STAT-3 pathways in early CRCs, indicating an important role of cytokines in mediating the crosstalk between stromal cells and CRCs. Interestingly, studies have also revealed that the activation of Notch signaling could enhance the production of IL-6 in stromal cells in the TME and then promote tumor cell growth and disease progression (97, 98). The study conducted by Jin et al. (99) indicated that the activation of Notch signaling was mediated by two components of the NF-κB cascade that could upregulate IL-6 expression in breast tumor cells. In turn IL-6 enhanced the activation of Notch again in an autocrine action pathway (100). Other studies reported that IL-6/Notch signals could cooperatively stimulate tumor metastasis and drug-resistance (101, 102). In addition, Wongchana and Palaga (103) found a direct regulatory effect of Notch on IL-6 activation at the transcript level in immune cells, and another study (104) found that IL-6 was also a mediator of crosstalk between fibroblasts and tumor cells in the CRC TME. Taken together, these results strongly suggest that IL-6 is a critical mediator of the interaction between CSCs and their surrounding cells within the TME.

### IL-8

Previous studies have suggested that IL-8 is an inflammatory cytokine released from many types of cells, such as macrophages, stromal, endothelial, epithelial and tumor cells, involved in promoting EMT, angiogenesis, tumor growth, metastasis and the immunosuppressive microenvironment in human cancers including CRC (105–112). Previous studies also revealed that a high expression level of IL-8 in human CRC tissues, mediated by IL-8 functional receptors IL-8RA and IL-8RB (43, 110, 113–115), was particularly associated with the properties and features of CSCs (51, 113). Recent studies have confirmed that IL-8 is an of the important modulators of the biological behavior of CSCs e.g., stemness properties that contribute to CRC recurrence and metastasis (42, 113–117). These regulatory effects were found to be mediated *via* a direct impact on the generation and maintenance of CSCs (42, 113–117) through its functional receptors IL-8RA and IL-8RB expressed in CSCs (43, 110, 113–115). A study by Hwang et al. revealed that the stemness properties of CD44-positive CSCs in CRC were regulated by Snail-IL-8 axis. Results from their works demonstrated a significant correlation between Snail and both IL-8 and CD44 expression; Snail and IL-8 are frequently expressed in CD44-positive CSCs, which have significant stemness properties and more malignant features. The authors further revealed that Snail could directly modulate IL-8 at the transcriptional level. Blocking IL-8 signaling by using shRNA or neutralizing antibody could significantly decrease the expression of stemness genes and reduce drug-resistance. Their findings provided direct evidence to show the modulatory effect of Snail and IL-8 on stem-like properties in CRC.

Luo et al. (118) also demonstrated that mono (2-ethylhexyl) phthalate treatment could remarkably increase the population of CSCs and promote the association of the β-catenin-TCF with the IL-8 promoter, both in cell lines and in mice. Chang et al. (119) demonstrated that Oct4 is a gene involved in the stemness of SCs and it is regulated by cytokines. They reported that IL-8 stimulated the expression of OCT4 and stemness properties in tumor cells (119). Fisher et al. (120) confirmed that targeting IL-8 signaling in the TME could remarkably decrease the tumor volume in mice with xenografts. They further showed that IL-8 stimulation induced a dose-response in *in vitro* CSCs, and blocking IL-8 or IL-8 receptor signals significantly inhibited cell cycle progression (cyclin D1 and B1) and induced a decreased rate of proliferation and angiogenesis in both *in vitro* CSCs and *in vivo* xenograft mice (120). Recently, Shimizu and Tanaka (121) showed that overexpression of IL-8 could induce increased glucose uptake, which was required for the generation and maintenance of CSC characteristics in colon cancer cells.

The effect of IL-8 produced by stromal cells on CSCs was also studied. Kim et al. (96) showed that IL-8 produced from stromal myofibroblasts could expand the population of CSCs by activating hes family bHLH transcription factor 1 in early CRCs, suggesting an interaction between CSCs and stromal cells. Recent evidence has shown that MSCs are an important source of IL-8, and MSC-derived IL-8 levels are even higher than CRC cell-derived IL-8 levels in CRC (122), which stimulates angiogenesis in a paracrine manner (113). Furthermore, the activation of the IL-8 network, modulated by a set of cytokines such as IL-1β (123), in the TME may contribute to the establishment of an immunosuppressive CSC niche (11, 113–115). In a recent study, we showed that the IL-8 niche of CSCs is regulated by IL-1β throughout the colorectal adenoma-carcinoma sequence (123). Interestingly, recent studies also reported (124) that IL-8-derived from CRC cells with high stemness features stimulated the process of angiogenesis. Therefore, this evidence suggests that crosstalk between CSCs and their surrounding cells contributes to tumor angiogenesis and progression, in which IL-8 functions as a key regulator of CSCs in CRC (113).

### IL-10

IL-10 is produced by many types of cells, including T cells, monocytes, macrophages, and epithelial cells. IL-10 exhibits a dual role, both promoting and protecting effects, in CRC development and progression. Studies have shown that binding of IL-10 to its receptor IL-10Rα results in the activation of signals e.g., STAT-1, STAT-3, and STAT-5, and then activates selected genes (125). Kang et al. previously reported that IL-10 promoted self-renewal of hematopoietic SCs (126). Recently, Biton et al. (127) found that intestinal SCs (ISCs) expressed several cytokine receptors including the IL-10 receptor IL-10Rα. They reported that both coculture with intestinal regulatory T cells (Tregs) and stimulation with IL-10 led to ISC expansion within organoids, and cells from IL-10-treated organoids had a significantly more “stem-like” pseudotime distribution (127). Their results indicated that IL-10 exhibits modulatory potential on ISC renewal. Since the modulatory mechanisms of cytokines on CSCs are similar to those on ISCs, we postulate that IL-10 may have a modulatory effect on CSCs. Future studies that identify the expression of IL-10Rα in pCSCs/CSCs and the exact effects of IL-10 on pCSCs/CSCs need to be performed.

### IL-17A

IL-17 is an inflammatory cytokine that released from numerous types of cells such as TH17 cells, gamma-delta-T cells (γδ/IL17 cells), Tregs, stromal cells, and tumor cells. Extensive studies have shown that IL-17A contributes to the initiation, progression, metastasis, and drug-resistance in diverse types of human cancers (69, 128). More recently, IL-17A has been implicated in the regulation of CSCs in cancers (83, 129). Previously, a study by Sui et al. (69) reported that IL-17A contributed to resistance to the chemotherapeutic drug cisplatin *in vivo*. Their results showed that IL-17 promoted the viability of HCT116 colorectal cells treated with cisplatin, while blocking IL-17 signaling downregulated apoptosis-related proteins and led to an increased rate of apoptosis in HCT116 colorectal cells.

Furthermore, Lotti et al. (130) found that IL-17A produced by tumor-associated fibroblasts in the CRC TME may drive the growth of CSCs. Their data showed that exogenous IL-17A enhanced CSC self-renewal and invasion, and targeting IL-17A signaling could significantly suppress CSC growth. They reported that fibroblasts in the TME were activated in response to chemotherapy in patients with CRC, which further resulted in increased production of IL-17A from activated fibroblasts and functioned as a mediator for the induction of drug-resistance. Therefore, they suggested that blocking IL-17A signaling may be a potential therapeutic strategy for overcoming drug resistance in CRC.

### IL-22

IL-22 is the main cytokine product of TH22 cells (53). The majority of studies showed a positive link between IL-22 and CRC initiation and progression (131–134). Recent scientific evidence suggests that the promoting effect of IL-22 on CRC development occurs *via* the induction of stemness in tumor cells (135). In line with this hypothesis, Xi et al. (136) demonstrated that IL-22 could stimulate the activation of STAT-3 and then upregulate the expression of PD-1 in human colon cancer cells. Since STAT-3 is highly expressed in CSCs, it is reasonable to speculate about potential modulatory effect of IL-22 on CSCs.

Furthermore, studies have reported that the promoting effect of IL-22 on CRC development occurs through the activation of STAT-3 (137), and neutralizing both IL-22 and STAT-3 signals reduces tumorigenesis *in vivo* and *in vitro* (61). Kryczek et al. (138) have previously shown that IL-22 could enhance CRC cell stemness and tumorigenic potential by upregulating core stem cell genes that relied on a STAT-3-dependent pathway, and pretreatment with anti-IL-22 antibody could significantly suppress tumor development and growth in mice after the subcutaneous injection of primary colon cancer cells. Lindemans et al. (139) further confirmed that recombinant IL-22 could directly increase proliferation and promote intestinal SC expansion through the activation of STAT-3 phosphorylation in LGR5-positive SCs and that this process was involved in intestinal epithelial regeneration.

OLFM4 is a member of the olfactomedin domain-containing protein family, and overexpression of OLFM4 has been identified in intestinal SCs (140). Recently, Neyazi et al. (141) reported that overexpression of OLFM4 in the colonic epithelium was modulated by IL-22. By using patient-derived colonic epithelial organoid cultures (EpOCs) model, they were able to show that IL-22 stimulation significantly upregulated the expression of OLFM4. Dame et al. (142) confirmed that OLFM4 in LGR5 positive pCSCs in the colonic adenomatous epithelium was modulated by IL-22. Their findings indicated that pCSCs are regulated by IL-22 in precancerous adenomas. Finally, studies indicated that IL-22 might also be implicated in drug resistance in both CRC patients (143) and CRC cell lines (144). Additionally, studies that focus on the contributory mechanisms of IL-22 to drug resistance in CRC are necessary.

### IL-23

IL-23 is an inflammatory cytokine mainly produced by TH cells, macrophages, and dendritic cells. In patients with adenoma and CRC, increased IL-23 has been detected and may function as an upstream factor to regulate the production of IL-17A from TH17 cells (145). A study by Wang et al. (146) showed that IL-23 stimulated the self-renewal of CD133 positive CSCs in ovarian cancer in an autocrine manner by activating the STAT-3 and NF-κB signaling pathways. In addition, IL-23 can activate stemness-relevant genes, such as the Wnt/Notch signaling pathway in esophageal carcinoma (147) and the STAT-3 signaling pathway in gastric cancer (148). Furthermore, a study by Chan et al. (149) showed that IL-23 was sufficient to induce colorectal tumorigenesis. Suzuki et al. (150) reported that IL-23 could directly stimulate the proliferative and invasive activities of CRC cells, and Zhang et al. (151) reported a selective effect of IL-23 on CRC metastasis both *in vitro* and *in vivo*; thus, all this evidence may suggest a direct or indirect effect of IL-23 on CSCs.

### IL-33

As reviewed by us and others, the contribution of IL-33 to the development and progression of CRC has been intensively studied (54, 152). Considerable evidence suggests that IL-33 promotes the development of CRC through several mechanisms, such as stromal remodeling, proangiogenesis, the induction of other protumor factors and immunosuppression (54). The modulatory effect of IL-33 on the properties of SCs has been reported. For example, Huang et al. (153) reported that hematopoietic SC regeneration after radiation injury could be stimulated by IL-33. Taniguchi and colleagues (154) reported that the formation of an IL-33–TGF-β niche was vital for the promoting effect of CSCs on carcinogenesis in a mouse model of squamous cell carcinoma, in which IL-33 stimulated the differentiation of macrophages and then sent TGF-β signals to CSCs to induce invasion and drug-resistance. Zhang et al.’s study showed that the serum level of IL-33 may serve as a predicator of cetuximab treatment efficacy in patients with CRC (72).

Regarding the role of IL-33 in colorectal adenomas, IL-33 signaling has been reported to promote intestinal polyposis *via* activation of the tumor stroma (155). More recently, Fang et al. (156) revealed a promoting effect of IL-33 on stemness properties in CRC. They showed that IL-33 significantly activated the core stem cell genes NANOG, NOTCH3, and OCT3/4 *via* the ST2 signaling pathway, activated the phosphorylation of c-Jun N-terminal kinase (JNK) and enhanced binding of c-Jun to the promoters of the core stem cell genes.

### IFN-γ

Several studies have shown that IFN-γ is a critical cytokine regulating CSC survival and stemness features (48, 157, 158). Ni et al. (48) reported that IFN-γ selectively upregulated the apoptosis pathway and promoted the apoptosis process by binding to receptors expressed in colon cancer LGR5-positive CSCs both *in vitro* and *in vivo*. In addition, the authors found that the administration of IFN-γ with the chemotherapy agent oxaliplatin resulted in a synergistic anticancer effect (48). Furthermore, a study by Song et al. (159) showed that non-small cell lung cancer-derived cell lines treatment with IFN-γ at a low dose induced a higher proportion of CD133 positive CSCs, and treatment with IFN-γ at a high dose resulted in enhanced apoptosis of tumor cells, indicating a modulatory effect of a low dose of IFN-γ on tumor cell stemness. More recently, Zhuang et al. (160) showed that IFN-γ at a dose of 26.68 ng/mL could remarkably inhibit ALDH1-positive CSCs and decrease the expression of ALDHA1 protein in breast cancer cells. Therefore, IFN-γ might be proposed as a selective anti-CSC cytokine, and modulating the IFN-γ response could function as a targeting strategy for CSCs in cancers (161). Indeed, we have previously found that the expression level of IFN-γ in the adenoma stage was slightly increased and then it was significantly decreased in the CRC stage, implying a selective role of IFN-γ in the management of malignant status along the adenoma-carcinoma sequence (47), in which increased IFN-γ in the adenoma stage maintains pCSCs in a relatively stable state, and decreased IFN-γ level in the CRC stage enhances CSC stemness and malignancy. In the future, the roles and exact mechanisms of IFN-γ as a key factor in modulating pCSCs/CSCs along the adenoma-carcinoma sequence need further exploration.

**Figure 1:**
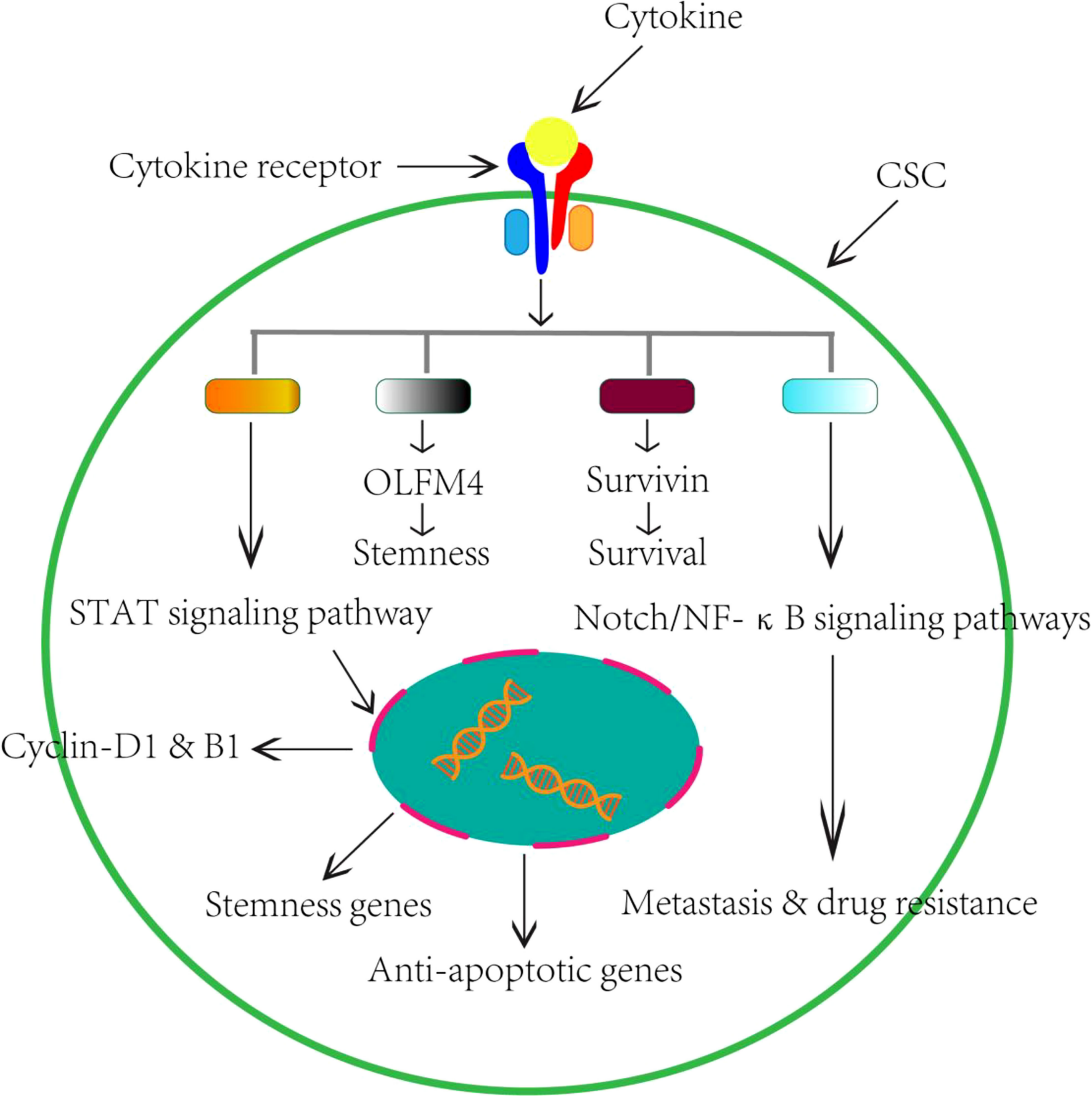
Schematic perspectives of potential role of cytokines on CSCs in the CRC. Based on current research findings, we therefore summarized the regulatory effects of cytokines on CSCs in the CRC.

Despite the complexity of the cytokine network, there are now considerable data suggesting the modulatory effect of cytokines on pCSCs/CSCs during the process of CRC (**Table 1**). Based on current findings, the possible regulatory mechanisms of protumor cytokines on CSCs are summarized in **Figure 1**.

**Table 1:** Main cytokines known to modulate CRC CSCs.

Cytokines		Main cellular sources		Target receptor		Effect on CSCs		Experimental model		References
IL-4		TH2 cells		IL-4Rα, IL-2Rγ		Inhibited CSC apoptosis		*In vitro*, *in vivo*		(79, 81)
IL-6		macrophages, T cells, adenoma/CRCcells		IL-6RA, gp130		Stimulated CSC phenotypes and properties by acting diverse signaling pathways		*In vitro*, *in vivo*, human biopsy		(66, 92, 93, 95)
IL-8		Macrophages, monocytes, T lymphocytes, endothelial cells, and adenoma/CRC cells		IL-8RA, IL-8RB		Stimulated CSC stemness, properties, generation, and maintenance; recurrence and metastasis		*In vitro*, *in vivo*, human biopsy		(24, 118–121, 124)
IL-10		Macrophages, Tregs, adenoma/CRC cells		IL-10Rα		Promoted stemness of ISCs, its effect on CSCs has not been reported		*In vitro*		(127)
IL-17A		TH17 cells and neutrophils, adenoma/CRC cells		IL-17RA, IL-17RB, IL-17RC		Stimulated CSC stemness, growth and drug-resistance		*In vitro*, human biopsy		(69, 130)
IL-22		TH22, TH17 cells		IL-22R		Enhanced CRC cell stemness and tumorigenic potential, activate stemness gene and increase drug-resistance		*In vivo*, *in vitro*, human biopsy		(138, 139, 143, 144)
IL-23		T lymphocytes, macrophages, and dendritic cells		IL-23R		Enhanced CSC self-renewal, proliferation, stemness		*In vitro*		(150)
IL-33		Epithelial, endothelial, fibroblasts, and adenoma/CRC cells		ST2, IL-1RAP		Promote CSC stemness properties and drug-resistance		*In vivo*, human biopsy		(72, 156)
IFN-γ		TH1 cells, cytotoxic T cells, NK cells and macrophages		IFNGR1 and IFNGR2		Enhanced CSC apoptosis, inhibit CSC differentiation		*In vitro*		(48)

## Clinical implications for CRC development, prevention, and treatment

### Could modulation of cytokine signaling affect the function of MSCs?

Published literature indicated that the interaction between MSCs and cytokines might create a favorable milieu to promote tumor angiogenesis, metastasis, drug resistance, and recurrence (122, 162, 163). Recently, we showed that TH17/IL-17A signal activation is associated with dynamic reaction of stromal cells including MSCs throughout the colorectal adenoma-carcinoma sequence (50), which has been verified as a promoting factor contributing to the adenoma and CRC progression (164). Thus, targeting cells or elements, e.g., fibroblasts, macrophages and MSCs and extracellular matrix (ECM) components within the TME, could remodel the CSC niche and this would affect CSC characteristics (165). For example, tumor stromal fibroblast-derived IL-8 stimulates the stemness properties in ovarian cancer cells (166). Similarly, tumor-associated macrophage-derived cytokines, such as transforming growth factor (TGF)-β, tumor necrosis factor (TNF)-α, IL-6 and IL-10, promote CSC transition and characteristics through epithelial–mesenchymal transition (EMT) in diverse types of cancers (167). Shintani et al. (168) reported that fibroblast-derived IL-6 mediated the communication between fibroblasts and cancer cells and contributed to chemoresistance by enhancing EMT in non-small-cell lung carcinoma. In breast cancer, blocking Hedgehog signaling in fibroblasts could restore the sensitivity of resident CSCs to chemotherapy agents (169). Stromal cells can also function as a vehicle to deliver cytokines. Experimental studies have shown that MSCs could be used as a cytokine delivery approach that provides a sustained release of target cytokines in the TME and leads to a more effective antitumor response (170). Therefore, the potential of MSCs as a vehicle for the delivery of bioagents that target cytokine signals into the CSC niche and have a modulatory effect on CSCs should be evaluated.

### Are cytokines involved in the modulation of pCSCs/CSCs in colitis-associated CRC?

Previously, Yasuda reported different rates of expression of the stem cell markers CD133, OCT4 and NANOG between CAC and sporadic CRC tissues (171). Iwaya et al. (172) found that the density of CSCs labeled by LGR5 in CAC was lower than that in sporadic CRC. However, Nakagomi et al. more recently reported that increased expression of CSCs is observed in both sporadic CRC (N=23) and CAC (N=22) neoplastic lesions. They found that the expression of CSCs labeled by ALDH1A1 in CAC tissues was higher in those with a longer disease duration than in those with shorter disease duration. CAC exhibits extensive and long-lasting chronic inflammation, and cytokines have been shown to be the most important driving mediators in the induction and maintenance of chronic inflammation (173). These results may imply a key role for cytokines in affecting CSCs in CAC.

Kazama et al. (174) showed that the rate of CD133-positive CSCs in CAC tissues was remarkably higher than that in dysplastic tissues, but the rate of LGR5-positive CSCs was not. Their results may suggest that pCSCs also exist in colitis-associated dysplasia.

Furthermore, their results explained the difference in the CSC-positive rate between different markers in CAC tissues. By using a mouse CAC development model induced by azoxymethane and dextran sodium sulfate treatment, Kim et al. (175) showed that CSCs in crypt base regions detected by RNA *in situ* hybridization in all dysplasia and CAC samples were increased. Carpentino et al. (24) used fluorescence-activated cell sorting to isolate and identify pCSCs labeled by ALDH from human colitis tissues and their transition to CSCs in both xenografting experiments and *in vitro*. They demonstrated that pCSCs/CSCs contribute significantly to colorectal carcinogenesis from colitis to CAC (24). Therefore, current scientific evidence suggests the involvement of pCSCs in the development of CAC. The precise modulatory effects of cytokines on pCSCs/CSCs throughout the inflammation-dysplasia-CAC sequence remain unclear, however, a hypothesis has been proposed (176) in which the promoting role of cytokines on pCSCs/CSCs should not be ignored.

### How does cytokines modulate pCSCs/CSCs in CRC with a familial adenomatous polyposis background?

Similar to sporadic adenoma polyps, several studies have identified an increased number and enhanced survival of pCSCs in FAP tissues (177, 178) (198). For example, Jennelle et al. (179) showed an increased rate of pCSCs positive for LGR5 in 7 subjects with Lynch syndrome, 4 subjects with FAP and 1 subject with MUTYH-associated polyposis syndrome, which were expressed in colon crypts of subjects with FAP. Ma et al. (180) reported that sulindac treatment of FAP patients might significantly change their pCSC dynamics, indicating that pCSC alterations may be a promising biomarker. However, since FAP is a rare disease, the modulatory effect of cytokines on FAP pCSCs/CSCs remains to be evaluated.

### Can selective reprogramming of the pCSC/CSC niche by engineering cellular or inflammatory components affect CRC initiation and progression?

In CRC prevention, one of the most important steps is to reduce the occurrence of precancerous adenomas. Clinically, the management of adenomas in patients relies on colonoscopy, during which the majority of adenomatous polyps can be removed. After the polyps are removed, patients, particularly elderly patients, are advised to undergo regular colonoscopy follow-ups to prevent subsequent CRC (181, 182).

Recent clinical trials have shown that chemoprevention is a promising method to reduce the occurrence of adenomas (183), and nonsteroidal anti-inflammatory drugs (NSAIDs) can significantly reduce the number and size of adenomas in patients with familial adenomatous polyposis (181). However, the effect is incomplete and it is unlikely to replace colonoscopic polypectomy as primary therapy. Furthermore, this NSAID-associated adenomatous polyp reduction cannot decrease the long-term CRC risk. Long-term treatment with NSAIDs may also increase the risks for severe side effects, such as cardiological toxicity (184), renal functional impairment (185) and gastrointestinal mucosa damage (186). For example, approximately 1 to 2% of patients experience a serious gastrointestinal complication during treatment with NSAIDs, which include mucosa severe mucosal damage, ulcers and bleeding (186). Therefore, there is an urgent need to seek novel preventive drugs that are more effective and safer.

**Figure 2:**
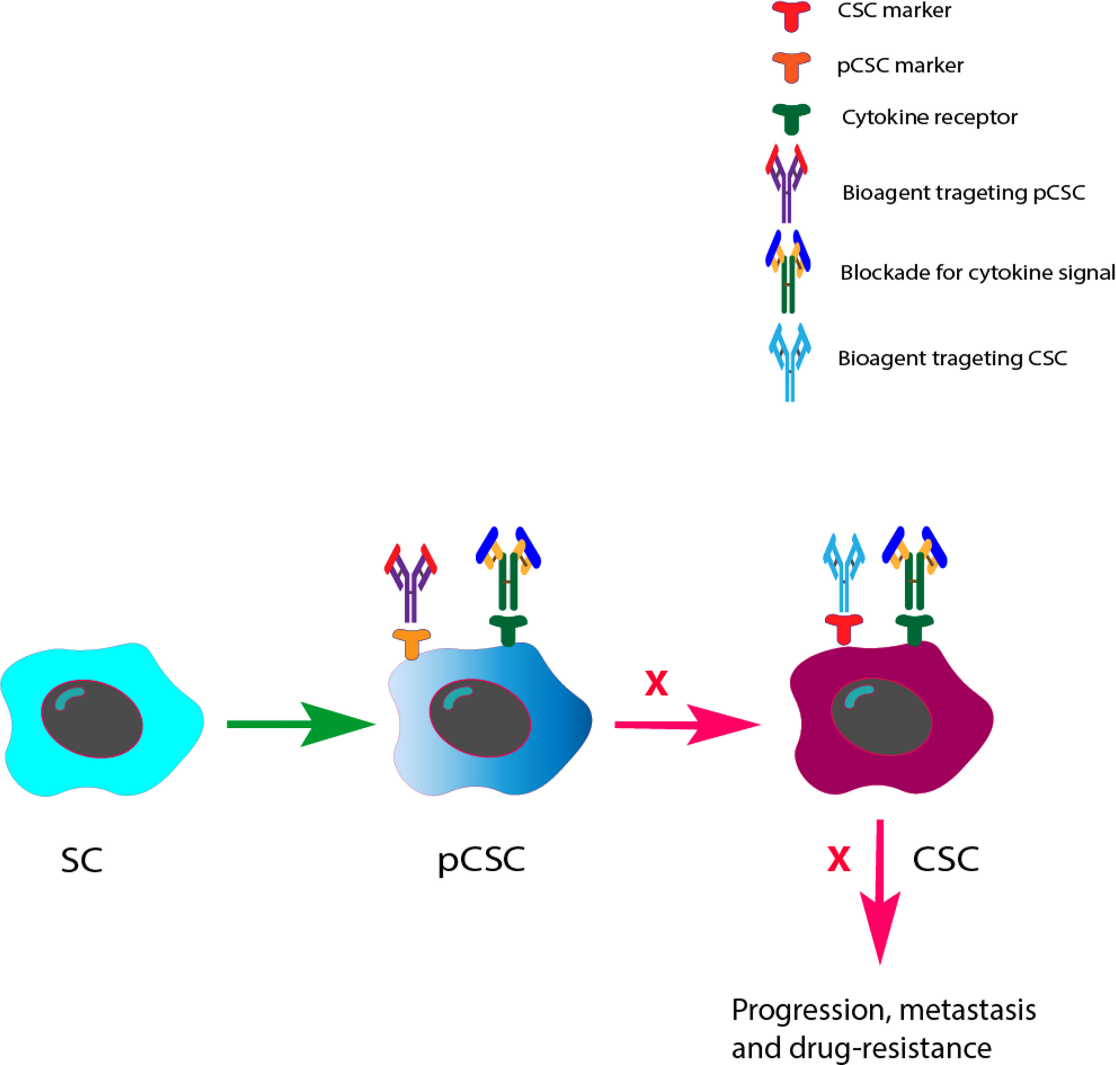
Future perspectives of cytokine signal blockades with pCSC/CSC target therapies in the prevention and inhibition of CRC development and progression. Based on the previous finding that pCSCs have an potential for bidirectional differentiation to both benign and malignant lesions, we therefore prospected that bioagents that can precisely target pCSCs/CSCs with cytokine signals may change both the differentiation direction of pCSCs at the precancerous stage and the effect of CSCs at the CRC stage.

It has recently been discussed that therapeutics that target pCSCs combined with their regulatory signals represent an attractive approach. Studies have revealed that pCSCs have the potential for bidirectional differentiation into both benign and malignant lesions, which depends on the interactions between pCSCs and their niche (6). Therefore, how to induce a pCSC to differentiate to a benign direction becomes important. Since cytokines have been reported to be modulators for CSCs, blocking cytokine signals within the niche may have an impact on the differentiation of pCSCs to CSCs and then affect the initiation of precancerous adenomas to CRCs. Several possible strategies need to be experimentally evaluated and addressed. For example, can we use blockades of cytokines combined with selective bioagents targeting pCSCs to modulate the malignant perspectives of a pCSC toward a CSC? Could pCSCs be reversible to SCs? Finally, the efficacy of cytokine signaling blockade combined with CSC targeted therapy in CRC needs to be assessed (refer to **Figure 2**). In the future, answers to these questions are important for understanding the mechanisms of the progression of a pCSC to a CSC and the identification of novel therapeutics to block this process.

As mentioned above, the biological behavior of pCSCs/CSCs in adenoma/CRC mass is modulated by their niche, which consists of distinct types of cells and factors. Therefore, selective reprogramming of the pCSC/CSC niche by engineering cellular or inflammatory components may affect the biological functions of pCSCs/CSCs (187). Indeed, Park et al. (188) demonstrated that caffeic acid could effectively inhibit self-renewal capacity, stem-like characteristics, and migratory capacity of CD44-positive and CD133-positive CSCs both *in vitro* and *in vivo*. Moreover, Park et al. (188) revealed that PI3K/Akt signaling might be linked to multiple CSC-associated characteristics, such as radioresistance, stem-like properties, and tumorigenic potential in CRC. Therefore, signaling pathways that regulate CSC biology and function may be identified as promising CSC targets in the future.

The SC stemness landscape is associated with the response to immunotherapy in CRC (35). Cytokines, as modulators of CSC stemness properties, may also serve as therapeutic targets in CRC. Indeed, previous studies have reported that increased expression levels of cytokines are associated with the initiation of adenoma and CRC in both Apc (Min/+) mice (143) and humans (51, 189), and blocking cytokines or their receptors by the administration of specific antibodies, e.g., anti-IL6 and anti-IL-17A/receptors, effectively suppresses the development and progression of CRC (190, 191). Recently, SC therapies that target CSCs or MSCs have been tested and have shown certain efficacy in established CRCs, which suggests that selectively targeting CSCs is a promising therapeutic strategy to eliminate the development of CRCs and reduce the risk of recurrence (37). Therefore, targeting cytokine signals that modulate CSC differentiation and function might represent an attractive and novel approach to combat CRC and improve the current CSC targeted therapy.

### Can targeting cytokine signaling improve drug sensitivity?

Drug-resistance is a major problem for cancer patients treated with chemotherapy. Studies have shown that CSCs function as a major driving force of drug-resistance and they are deeply involved in the pathogenesis of drug resistance (192, 193). Targeting cytokine/receptor signals holds promise for overcoming drug resistance and restoring sensitivity to anticancer therapies (77, 194). For example, IL-4 has been reported to protect CRC CSCs from apoptosis and the potential benefits of standard chemotherapies, radiotherapies or immunotherapies in combination with IL-4 inhibitors may enhance the therapeutic efficacy in the context of CRC (77).

A study by Jiang et al. (194) assessed the efficacy of targeting IL.-8 signaling to overcome drug resistance in advanced gastric cancer, and their results showed that increased expression of IL8 significantly activated drug resistance. IL8 signaling targeted by RNA interference or reparixin reversed chemotherapy resistance with limited toxicity *in vivo* and *in vitro*. Targeting CSCs has been recognized as an effective therapeutic for CRC (37). Studies have revealed that the effect of IFN-γ on CSCs was dose-dependent (159). In non-small cell lung cancer-derived cell lines, IFN-γ at a low concentration induced enhanced stemness, and IFN-γ at a high concentration caused apoptosis in CSCs, which could improve the therapeutic efficacy of chemotherapeutic drugs (48, 159). This bidirectional effect of IFN-γ on CSCs may suggest a possible strategy for modulating IFN-γ signaling to selectively inhibit CSC survival and stemness.

However, how to reduce the side effects of cytokine-based immunotherapy is still a challenging issue (195). Most cytokines exhibit a pleiotropic effect and work in a complex network. They play a significant role in immune regulation, antimicrobial infection, and modulating the body’s response to disease. Systematic blockade or enhancement of cytokine signaling may significantly influence host immune homeostasis. In addition, bioagents that target cytokine signals may have their own side effects and drug toxicity (195). Therefore, a better approach for cytokine-based therapy would be the precise targeting of local cytokine signals that can be reached by a selective delivery vehicle (170).

## Conclusions

In light of the current knowledge, we may conclude that cytokines function as the main communicators to mediate crosstalk between pCSCs/CSCs and their niche, which is involved in both adenomatous polyp and sporadic CRC onset and progression. Additional studies focused on the pathways of pCSCs/CSCs regulated by cytokines could reserve new perspectives in the field of precision cancer-targeted therapy. In the future, more attention should be given to evaluating the mechanisms of cytokines in regulating the progression of pCSCs to CSCs in adenomas that hold promise for the design of novel therapeutics to prevent the onset of CRC. Finally, pCSCS/CSCs, together with cytokine signals, are attractive potential targets for the treatment of CRC. The therapeutic efficacy of targeting cytokine/receptor signals to inhibit the progression of pCSCs to CSCs is waiting to be evaluated in the future.

## Author contributions

GC had the idea for this review and performed the electronic search for literatures, ZW and HL performed literature selection, data extraction and analysis. ZP joined the data analysis and discussion. All authors contributed to the article and approved the submitted version.

## Funding

This study was supported by the grants from National Natural Science Foundation of China (81071969) and Medical Research Program, Northern Norway Regional Health Authority (SFP-44-04). The funder did not play any role in paper design, data collection, data analysis, interpretation, writing of the paper.

## Conflict of interest

The authors declare that the research was conducted in the absence of any commercial or financial relationships that could be construed as a potential conflict of interest.

## Publisher’s note

All claims expressed in this article are solely those of the authors and do not necessarily represent those of their affiliated organizations, or those of the publisher, the editors and the reviewers. Any product that may be evaluated in this article, or claim that may be made by its manufacturer, is not guaranteed or endorsed by the publisher.

## Abbreviations

ALDH1, aldehyde dehydrogenase 1; ALDH1A1, aldehyde dehydrogenase isoform 1A1; CRC, colorectal cancer; CSCs, cancer stem cells; ECM, extracellular matrix; EMT, epithelial–mesenchymal transition; EpOCs, epithelial organoid cultures; IFN, interferon; IL, interleukin; ISCs, intestinal stem cells; LGR5, leucine-rich repeat-containing G-protein-coupled receptor 5; MSCs, mesenchymal stem cells; NSAIDs, nonsteroidal anti-inflammatory drugs; NF-κB, nuclear factor Kappa B; OLFM4, olfactomedin-4; pCSCs, precancerous cancer stem cells; SCs, stem cells; STAT, signal transducer and activator of transcription; TGF-β, transforming growth factor-β; TNF-α, tumor necrosis factor-α; Tregs, regulatory T cells.
